# SARS-CoV-2 Spike Protein Post-Translational Modification Landscape and Its Impact on Protein Structure and Function via Computational Prediction

**DOI:** 10.34133/research.0078

**Published:** 2023-03-08

**Authors:** Buwen Liang, Yiying Zhu, Wenhao Shi, Can Ni, Bowen Tan, Shaojun Tang

**Affiliations:** ^1^ The Hong Kong University of Science and Technology (Guangzhou), Guangzhou, China.; ^2^ Analysis Center, Chemistry Department, Tsinghua University, Beijing, China.; ^3^ The Hong Kong University of Science and Technology, Hong Kong SAR, China.

## Abstract

To elucidate the role of post-translational modifications (PTMs) in severe acute respiratory syndrome coronavirus 2 (SARS-CoV-2) spike protein’s structure and virulence, we generated a high-resolution map of 87 PTMs using liquid chromatography with tandem mass spectrometry data on the extracted spike protein from SARS-CoV-2 virions and then reconstituted its structure heterogeneity caused by PTMs. Nonetheless, Alphafold2, a high-accuracy artificial intelligence tool to perform protein structure prediction, relies solely on primary amino acid sequence, whereas the impact of PTM, which often modulates critical protein structure and function, is much ignored. To overcome this challenge, we proposed the mutagenesis approach—an in silico, site-directed amino acid substitution to mimic the influence of PTMs on protein structure due to altered physicochemical properties in the post-translationally modified amino acids—and then reconstituted the spike protein’s structure from the substituted sequences by Alphafold2. For the first time, the proposed method revealed predicted protein structures resulting from PTMs, a problem that Alphafold2 has yet to address. As an example, we performed computational analyses of the interaction of the post-translationally modified spike protein with its host factors such as angiotensin-converting enzyme 2 to illuminate binding affinity. Mechanistically, this study suggested the structural analysis of post-translationally modified protein via mutagenesis and deep learning. To summarize, the reconstructed spike protein structures showed that specific PTMs can be used to modulate host factor binding, guide antibody design, and pave the way for new therapeutic targets. The code and Supplementary Materials are freely available at https://github.com/LTZHKUSTGZ/SARS-CoV-2-spike-protein-PTM.

## Introduction

RNA nonsynonymous mutations and amino acid post-translational modifications (PTMs) of the severe acute respiratory syndrome coronavirus 2 (SARS-CoV-2) spike protein have been shown to be 2 key contributors to the coronavirus disease 2019 pandemic [1–3]. There are numerous characterizations of different RNA variants on the virus' virulence and functions [3–7]; however, how different PTMs influence the virus’ structure, function, and virulence is much less studied [6]. Studies show that the protein’s structure and function are closely modulated by PTMs from lower-level organisms (such as viruses) to human and consequently play an important role in protein–protein interaction and signaling pathways [8–10]. The alterations of amino acid side chains resulting from PTMs without altering the primary amino acid sequences lead to intriguing structural and functional diversity and are, therefore, a delicate mechanism to maintain cellular protein homeostasis and execute complicated functions [2,11].

Spike protein PTM is a shared feature in different variants of SARS-CoV-2, which has been explored and whose importance has been proven in a few functional studies [1,12–14], as indicated by different PTM modification types, modified residues, and modification frequencies [1,13], as well as virus–host interaction sites, binding affinity, and virulence [12,14]. In particular, the spike protein PTM profile may reflect virulence and binding affinity with human host factors, leading to the hypothesis that types and distributions of spike protein PTM may uniquely define the virulence–host factor interaction [4,12,15]. Notably, the virus’ entry into the susceptible host cell is mediated by the interaction of the spike protein in the S1 subunit and cellular angiotensin-converting enzyme 2 (ACE2), a complex process requiring proteolytic activation and receptor binding [12,15–18]. In addition, both ASGR1 and KREMEN1 can also serve as SARS-CoV-2 receptors and might impact viral target cell range and antibody-mediated neutralization [19,20]. In summary, these studies highlight the fact that the spike protein is the most attractive immunogen for antibody production and vaccine development.

Unfortunately, existing studies mostly focus on characterizing RNA mutations in the spike protein that host factors and antibodies recognize, whereas the impact of PTM, which often modulates critical protein functions, is much less studied [3,5,7,21]. In fact, PTMs are key regulators of protein folding and they mediate protein interaction, stability, localization, and turnover [8–10]. For example, acidic phosphorylated residues can function as nucleation sites for the proper folding of the protein with hydrogen bounding that stabilize local structures [22]. In addition, some PTMs result in irreversible changes such as proteolysis and proteolytic cleavage of substrates, whereas aberrant PTMs cause protein misfolding and dysfunction, resulting in diseases such as Alzheimer’s disease [21].

Current protein structure characterization approaches such as cryogenic electron microscopy (cryo-EM) or computational prediction can neither accurately nor robustly capture the impact of various PTMs on the SARS-CoV-2 spike protein structure and interaction with host factors [9,23]. On the one hand, although cryo-EM studies had revealed distinct spike protein structures isolated from different viral strains, which are useful to identify strain-specific novel binding sites for antibodies and other reagents [24,25], it is impossible to identify the full spectrum of dynamic, post-translationally modified amino acids due to varying physicochemical conditions. On the other hand, cryo-EM crystal structural characterization is extremely time-consuming and costly, making it difficult to generalize in routine studies. Fortunately, with the advancement of the deep neural network algorithm, the computational prediction of protein structures enabled by Alphafold2 with a deep learning architecture showed comparable accuracy with cryo-EM using only primary amino acid sequences, and this process significantly reduced the amount of time and cost. However, Alphafold2 relies entirely on primary amino acid sequences and completely ignores the impact of PTM on protein structure formation [9,23,26,27]. Therefore, without a proper consideration of PTMs, Alphafold2’s predicted protein structure relying on primary sequences alone is not only biased but also misleading. Apart from this, although some studies also used computational approaches or dynamics simulations to model the structure of the spike protein and its binding affinity with the receptor ACE2 [5,28–31], there are no effective pipelines to study the impact of PTMs on the binding of the spike protein to host factors such as ACE2 [9].

**Fig. 1.**
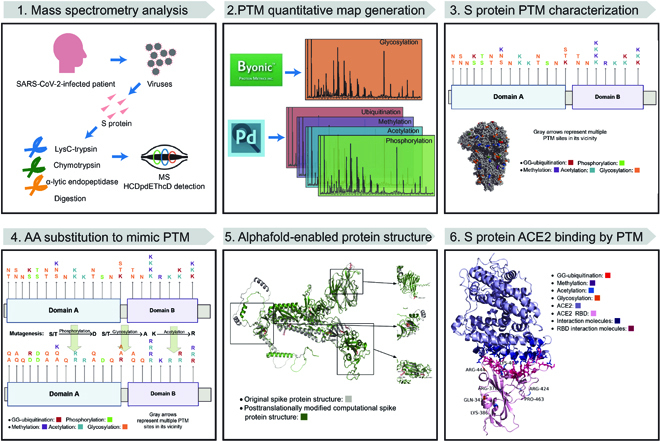
The workflow of SARS-CoV-2 spike protein PTM landscape construction and Alphafold2-enabled structure prediction. (1) LC-MS/MS data analysis and data collection. (2) PTM landscape construction. (3) Alphafold2 in silico structure prediction. (4) Mutagenesis approach on amino acid replacement. (5) Evaluation of PTM’s impact on protein structure compared to raw unmodified spike protein. (6) Simulation of ACE2 binding to spike protein’s RBD region with PTMs.

Here, we first generated a high-resolution quantitative PTM map on the SARS-CoV-2 spike protein using liquid chromatography with tandem mass spectrometry (LC-MS/MS) data to elucidate the comprehensive PTM sites, which was the first of its kind for SARS-CoV-2 studies. Then, computational predictions of post-translationally modified spike protein structures were postulated through Alphafold2 via in silico mutagenesis, a popular strategy to substitute a post-translationally modified amino acid with another amino acid to mimic the altered physicochemical feature. Mutagenesis is unarguably one of the most common methods to study PTMs [8,32]. For example, to mimic phosphorylation modification, the conventional mutagenesis strategy aims to replace serine or threonine with asparagine or glutamine, the negatively charged amino acids. Amino acid site-directed mutagenesis has revolutionized the understanding of structure and function of post-translationally modified proteins [8,33]. Our innovative use of the mutagenesis approach to evaluate the post-translationally modified amino acids allows us to study the protein structure via Alphafold2 without needing to perform cryo-EM, a costly and complicated method requiring expensive instruments, while retaining high structure prediction accuracy. Furthermore, we evaluated the heavily modified receptor-binding domain (RBD) region of the spike protein and its interaction with ACE2 to reveal the strength of the binding, which may provide scientific insights into the study of the viral spike protein structure and the design of more effective therapeutic approaches. The workflow of the study is shown in Fig. 1.

## Results

In this study, we generated a high-resolution quantitative map of 87 PTMs on the spike protein from LC-MS/MS data and proposed an in silico amino acid substitution to mimic the altered physicochemical properties of PTM sites, and then reconstituted the spike protein structure using Alphafold2. Through this study, we identified the role of PTMs and revealed the structural heterogeneity of different PTMs in the SARS-CoV-2 spike protein. In addition, we conducted protein–protein interaction studies to understand how PTMs impact the spike protein’s interaction and binding with host factors such as ACE2.

### High-resolution quantitative map of PTMs in the spike protein

MS-based proteomics using tandem mass spectrometry makes it possible to identify many types of PTM information and construct the PTM landscape. Peptides with different modifications produce varying mass shifts in spectra, which allow for reliable computational predictions of PTMs. To automatically assign peptides with modifications from MS spectra, database search tools, such as Proteome Discoverer, had been developed. During database search, possible modified forms of each peptide are matched to a protein database with peptide spectra information. However, the search space grows exponentially as the number of possible modifications increases; thus, only a limited number of modification types could be computed in one round of analysis in most cases. To date, there are a few studies on SARS-CoV-2 spike protein PTMs, and one example is the characterization of spike protein glycosylation [1]. Nevertheless, a complete landscape of widely studied modifications for spike protein is still lacking. To construct a comprehensive PTM map of the spike protein, we here performed a heuristic database search for diverse PTMs based on MS raw files of viral spike proteins isolated from infected patients [1]. Besides glycosylation, which has been proven to affect the structure and functions of the spike protein, we identified a total of 87 PTMs, including phosphorylation, acetylation, methylation, and ubiquitination. This is so far one of the most comprehensive studies to obtain spike protein PTM information.

**Fig. 2.**
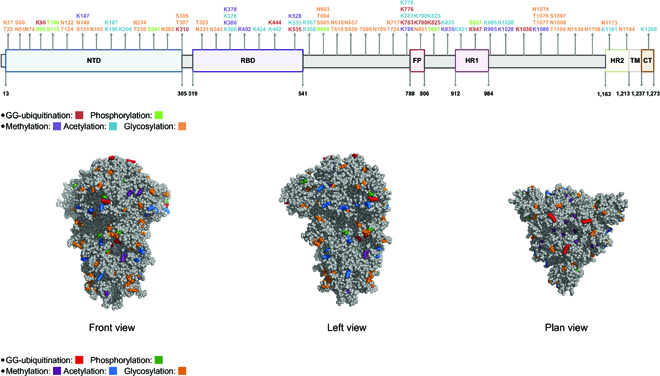
The distribution of 87 PTM sites from 5 major modifications on the SARS-CoV-2 spike protein. (A) A schematic illustration of the PTM site in the spike protein. (B) Structure-based display of PTMs in the spike protein. PTMs are marked on the top and side views of the structure of the trimeric SARS-CoV-2 spike protein.

The SARS-CoV-2 spike protein has a total of 1,273 amino acids, including a signal peptide (amino acids 1 to 13) located at the N-terminus, the S1 subunit (14 to 685 residues), and the S2 subunit (686 to 1,273 residues). In particular, there are 2 important domains in S1: an N-terminal domain (NTD), from residues 14 to 304, and the RBD, from residues 319 to 541. Apart from that, the transmembrane S2 subunit is composed of the fusion peptide (FP) (788 to 806 residues), heptapeptide repeat sequence 1 (HR1) (912 to 984 residues), HR2 (1,163 to 1,213 residues), transmembrane domain (TM) (1,213 to 1,237 residues), and cytoplasm domain (CT) (1,237 to 1,273 residues). Overall, quantitative analysis of LC-MS/MS raw files of the spike protein identified (a) the types of PTMs, (b) PTM localization, and (c) PTM frequency. In particular, PTM with a frequency of at least 40% (40% of the samples carrying the same modification for a given modified amino acid) was used for PTM identification. In total, we shortlisted 87 distinct PTMs on 1,273 amino acid residues across all 6 samples and fractions, accounting for 6.83% of the entire length, while there were 67 residues carrying a single modification and each of the remaining 20 residues has at least 2 different modifications, as shown in Fig. 2, Table 1, and Table S1.

**Table 1.** The S protein-altered protein amino acid sequence summary table based on “mutagenesis” amino acid substitution rules on the 87 PTMs.

PTMs	Amino acid	Substitution amino acid	Numbers	S1-non-RBD	S1-RBD (319 to 541)	S2 (686 to 1,273)
Glycosylation	Asparagine, N	Glutamic acid, Q	22	11	2	9
Serine, S/ Threonine/T	Alanine, A	18	11	1	6
Ubiquitination	Lysine, K	Arginine, R	10	2	2	6
Phosphorylation	Serine, S/ Threonine/T	Aspartic acid, D	7	5	0	2
Methylation	Lysine, K	Arginine, R	8	1	3	4
Arginine, R	Lysine, K	2	0	1	1
Acetylation	Lysine, K	Arginine, R	20	5	5	10
Total (overlapping PTM sites)			87 (20)	35 (0)	14 (6)	38 (14)

To summarize, the largest number of modified sites was found in glycosylation with 39 residues, followed by acetylation sites with 21 modified amino acids. In contrast, other types of modification such as phosphorylation occurred in less than or equal to 10 amino acids. It is widely believed that glycosylation is associated with a wide range of spike protein functions, including regulating viral tropism, protein stability, and shielding the underlying epitopes from immune surveillance [1]. Furthermore, it is known that the spike protein RBD region binds to the human host cell receptor ACE2 and mediates viral invasion [15–18]. In this study, we identified 14 PTMs in the RBD region of the spike protein S1 unit, including 3 glycosylation sites, 4 methylation sites, 2 ubiquitination sites, and 5 acetylation sites. We believe that these sites might have an important role in protein structures and contribute to the virus and host factors’ binding specificity and affinity during infection.

In general, PTM frequency is crucial to understanding the modification mechanism of each PTM. Although most PTM sites (67 modified amino acids) are modified by a single type of PTM, other PTM sites (20 modified amino acids in total), such as 378K, are not only methylated but also acetylated. For these 20 residues carrying multiple modifications, we discovered that only one amino acid was both phosphorylated and glycosylated, while the other 19 PTM sites were both phosphorylated and acetylated, which led to the neutralization of the amino acid's positive electrostatic charge. For instance, lysine acetylation enables the generation of novel recognition surfaces for the binding of proteins having “reader” domains specific for the post-translationally modified residues [34]. In summary, a comprehensive characterization of the PTM landscape permits a deeper understanding of the host factor recognition and binding, which further enables us to deeply understand the mechanisms of virulence and infectivity.

### Comparison of protein structures from computational prediction and cryo-EM experiment

To evaluate and validate the accuracy of the predicted spike protein structure, we compared its computational structure to cryo-EM’s experimental structures. First, for each PTM type, we performed site-directed amino acid substitution according to the substitution rule (Table 1), followed by computational prediction using Alphafold2 to model the spike protein’s structures based on the substituted sequence. Second, we aligned Alphafold2’s predicted structure with cryo-EM experimental structures to reveal structural similarities and differences. Last, we conducted PTM site substitutions on selected RBD regions and on the entire spike protein, and performed a head-to-tail comparison with the cryo-EM crystal structure. The resulting .pdb files are stored in Supplementary Materials.

**Fig. 3.**
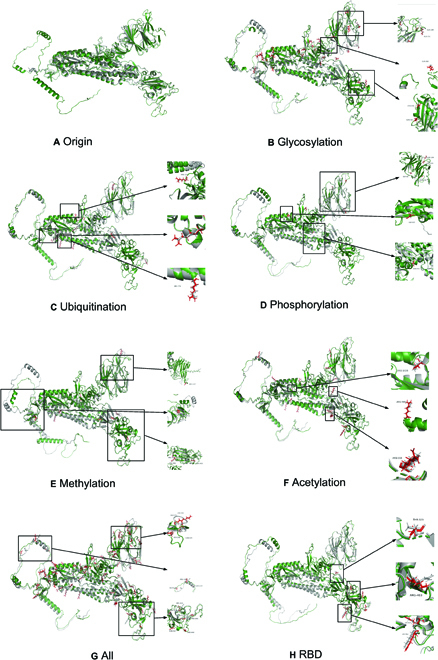
The alignment and its specific variants in each substitution site of individual computational structures. (A) Origin vs. cryo-EM experiment. Sliver represents the cryo-EM experiment structure, and green represents the original structure. (B to H). Sliver represents the original structure, and green and red represent the modified structures. (B) Glycosylation vs. origin. (C) Ubiquitination vs. origin. (D) Phosphorylation vs. origin. (E) Methylation vs. origin. (F) Acetylation vs. origin. (G) All vs. origin. (H) RBD vs. origin.

Alphafold2 computed an important score, the model confidence score, to indicate the fitness of the computational prediction. This score is crucial to evaluate the soundness of the prediction based on primary amino acid sequence alone. Thus, we compiled a summary of the confidence scores for each sequence carrying a different modification type, as shown in Table 2. Overall, we found that the computational structures had a high average confidence level from 76 to 78, which is a good confidence score indicating a good fitness during model construction, with essentially no marked difference from the cryo-EM structure. After that, we analyzed the predicted local distance difference test (pLDDT) score for each amino acid. Specifically, the spike protein backbone sequence, especially the α-helix, β-fold, and β-turn secondary structures, has a very high confidence level (>90). These high-confidence structures are mainly located in RBD, FP, and HR1 areas, which are responsible for the crucial functions of the spike protein. For instance, the RBD plays a vital role in virus infection by binding the ACE2 [15–18]. Apart from that, the confidence scores in NTD, HR2, TM, and CT areas are in range of 70 to 90, reflecting the immunologically important variation and other functions [35]. In contrast, there are very few regions with a weak reliability score of below 70. Notably, some amino acids are mainly located in the boundary of 2 adjacent functional domains, and others are in the N-terminal region, such as the amino acid from sites 1 to 14. Interestingly, some of these amino acids, such as 70 to 80 and 677 to 689, did not fall on domains such as α-helix or β-sheet in the cryo-EM structure. Finally, we compared the pLDDT scores for all amino acids between unmodified and post-translationally modified spike protein predicted structures (Fig. 3). Together, these results revealed a sequence-wise similarity in Alphafold2 model confidence scores between the unmodified and modified spike protein, especially on important domains of the protein structure bearing high confidence scores.

**Table 2.** The Alphafold confidence score and structural similarity comparison of the 5 major amino acid substitution rules for spike protein PTMs.

Type	Confidence	Proportion of modification sites	RMSD (origin)	RMSD (6VSB)	RMSD (6VXX)	TM score
Original	78.04		\	1.266	1.124	0.964
Glycosylation	77.64	3.14%	0.255	1.367	1.224	0.952
Ubiquitination	77.84	0.79%	0.163	1.311	1.141	0.966
Phosphorylation	77.84	0.55%	0.281	1.273	1.163	0.950
Methylation	77.55	0.79%	0.671	1.388	1.385	0.916
Acetylation	78.01	1.57%	0.354	1.315	1.224	0.963
Only RBD area	78.01	1.10%	0.349	1.305	1.328	0.945
All	77	6.83%	0.457	1.376	1.238	0.962

In this work, we further compared the predicted structure resulting from PTMs with the cryo-EM experimental structure. To begin with, we aligned the post-translationally modified computational structures with both 6VSB and 6VXX, the 2 earliest and highest-resolution cryo-EM experimental structures, and calculated the root-mean-square deviation (RMSD) and TM scores [36], as shown in Table 2. Second, we compared the post-translationally modified predicted structures with the unmodified predicted structures. Results showed that the RMSD for cryo-EM experiments (6VSB and 6VXX) ranged from 1.1 to 1.4. Based on these observations, we concluded that Alphafold2's prediction structures have remarkably high prediction accuracy and reliability, which can thus be used as a prediction tool for the PTM structures.

### Structural characterization of post-translationally modified spike protein on different modification types

To begin, we evaluated the impact of one PTM modification type by substituting amino acids with one modification at a time, for all PTMs types including phosphorylation, acetylation, methylation, glycosylation, and ubiquitination. We discovered that, although some modification types only occurred in approximately 1% of all amino acids, these PTMs significantly affected the protein structure and functions (Table 2). In fact, we noticed that different PTMs showed distinct impacts on the structure of spike proteins. First, we found that the most dramatic structure change was caused by methylation, with an RMSD score of 0.671, despite the fact that methylated amino acids only occupied 0.79% of the total sequence, only at a moderate modification frequency. Second, amino acid acetylation changed the predicted structures considerably, with an RMSD of 0.354. Third, both methylation and acetylation modifications on the amino acid lysine were involved in the neutralization of the positive electrostatic charge, and they contributed significantly to structural changes [34,37]. Finally, in contrast, a small RMSD score from both ubiquitination and glycosylation modifications indicated that these 2 modification types did not have a significant impact on protein structure, although these 2 modification types occurred with the highest frequency at 3.14% and 0.79% of amino acids of the total sequence, respectively. Particularly, glycosylation modification, despite having the greatest number of amino acids, had a minor impact on structures of important functional domains, as shown in Fig. 3. Nevertheless, glycosylation changed the protein’s structure in regions that were not crucial for spike protein binding [1]. Together, we proved that the number of total modification amino acids is not the deterministic factor for protein structure change; rather, the type of modification and the location of modified amino acids are more crucial for structural changes.

Next, we plotted the sequence-wise modified amino acids based on the mutagenesis substitution rule for each type of PTM modification in Fig. 3. Analyses showed that some modified amino acids did not directly lead to observable changes in the predicted spike protein structure. For example, arginine at position 1028 modified by glycosylation did not result in protein structure change, while the arginine at position 986 changed the structure considerably. Furthermore, several PTMs did not affect the overall structure of the spike proteins based on in silico amino acid substitution. Nevertheless, to our surprise, although some modified amino acids did not change the substructure in the modified residues’ surrounding regions, they indirectly influenced the distal regions’ substructure as shown in Fig. 3. In addition, for each amino acid carrying 2 modifications, we showed that lysine acetylation typically has a stronger impact on the structural changes compared to other types of PTMs.

To elucidate the role of the entire PTM landscape, we generated a computational predicted spike protein structure using all 87 PTM sites (regardless of the PTM types) via the site-directed amino acid substitution rule and then compared it to the predicted structure from the unmodified primary amino acid sequence. Basically, there was no marked difference between the spike protein’s unmodified structure and modified structure, especially for functional domains in the S1 and S2 region. Nevertheless, several PTMs slightly influenced the substructures in important regions, such as RBD. Interestingly, when we replaced all 87 amino acids carrying modifications, the resulting predicted spike protein did not end up with a structural change as much as focusing on methylation modification alone, although only 10 amino acids were affected by methylation. Thus, we concluded that different modification types have notably varying degrees of influence on protein structure, and we determined that methylation had the most profound effect on protein structure, with arginine at amino acid positions 147 and 386 contributing the most to structural changes, whereas most other modification types did not significantly alter protein structure in important functional domains, as shown in Fig. 3G.

Last but not least, to examine which PTMs were most strongly associated with the structural change in the RBD region, we evaluated the spike protein structure using PTMs only occurring on amino acids in the RBD region. The results showed that a total of 14 PTM sites in the RBD region resulted in considerable changes in the predicted spike protein structure with an RSMD value of 0.349. In summary, our results shed light on a systemic characterization of the important role of PTMs on the structure and function of the spike protein. Nevertheless, the interplay of different modification types in influencing the protein structure was very complex, and we postulated that the aggregated structural change is not simply an addition of each modification type; thus, further experimental validations are needed to uncover the underlying regulating mechanisms.

### Computational prediction of PTM on the binding affinity between SARS-CoV-2 spike protein RBD region and human host factors

**Fig. 4.**
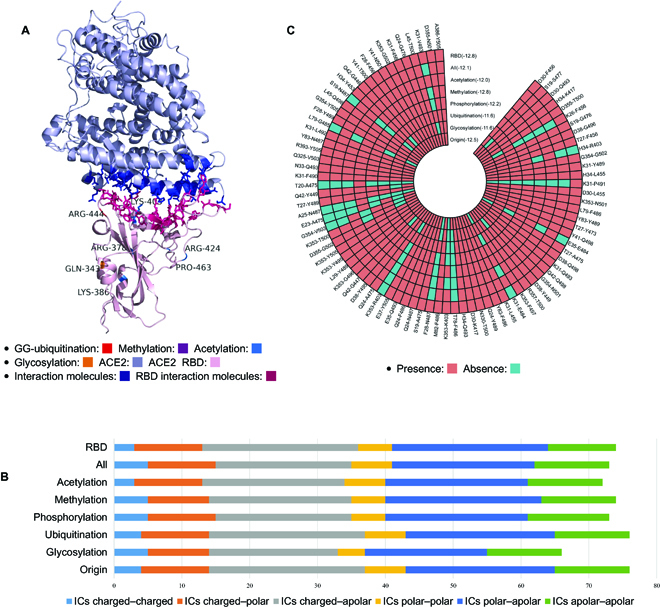
Binding affinity and interaction sites for the post-translationally modified spike protein and ACE2. (A) Structures of the post-translationally modified RBD-binding domain in complex with its receptor ACE2 and intermolecular interactions. Pink represents the RBD of spike protein, and light blue represents the ACE. (B) The number of different types of intermolecular contacts between the post-translationally modified spike protein and ACE2. (C) The intermolecular interactions between ACE2 and the RBD region in the post-translationally modified spike protein and binding affinity. Light blue represents the presence of interactions, and orange represents the absence of interactions.

As is known, the SARS-CoV-2 virion’s entry into host cells is mediated by its spike glycoprotein, which is mainly involved in communicating with human host factors such as the ACE2 receptor. Besides, ASGR1 and KREMEN1 have also been reported to be involved in SARS-CoV-2’s ligand–receptor interactions [19,20]. Here, we used computational approaches to analyze the virion spike protein and host factor binding interaction by evaluating the impact of PTMs as shown in Fig. 4.

Considering only the amino acids in the RBD, we systematically evaluated the impact of PTMs on RBD and the ACE2 binding complex in great detail (Fig. 4A). We found that the RBD’s PTMs did not result in marked structural changes in the spike protein and the ACE2 binding complex; however, we observed a number of PTMs outside the spike protein’s RBD that led to subtle structural changes in the interaction complex, which indicated that PTMs may indirectly exert their structural influence on amino acids that were located far away from the RBD binding region.

In an attempt to identify different intermolecular contacts (ICs) between the post-translationally modified spike protein and ACE2, we systematically analyzed the polar, apolar, and charged properties of the interacting residues. Results showed that the IC scores of residues in the binding domains were rather similar (from 70 to 75). However, we also found that the number of ICs for predicted structures was generally higher with PTMs (excluding ubiquitination) compared to predicted structures without any PTM, as indicated by the gaps between charged–apolar structures, as shown in Fig. 4B.

To demonstrate the ICs in greater detail, we wanted to see if binding sites for spike protein RBD and ACE2 exist. Figure 4C summarizes the unmodified and modified sequences (a total of 7 PTM sites) on the spike protein RBD with ACE2. The results showed that as a result of amino acid modifications, the spike protein RBD region and ACE2 interacting protein complex changed significantly.

In summary, we found that the highest spike protein RBD region and ACE2 binding affinity was achieved by methylation modification, with a PRODIGY binding score of −12.8, while the binding score for ubiquitination and glycosylation was at−11.6. In particular, some ICs were only found in one form of binding complexes. For instance, T78-F486 was only found in the original unmodified structure, whereas T20-A475 was only found in the predicted structure influenced by methylation. Apart from that, there were also binding sites carrying more than 2 modifications (Fig. 4). Besides the ACE2 host factor, we also calculated the binding affinity of the post-translationally modified spike protein to 2 other host factors: ASGR1 and KREMEN1 (Table S2). Results demonstrated that the spike protein bound to both ASGR1 and KREMEN1 with a higher affinity, which further validated that these 2 proteins are involved in SARS-CoV-2 virus–host interactions. The spike protein binding affinity with KREMEN1 (−9.1 to −10.6) was much higher than that with ASGR1 (−7.7 to −9.4). In conclusion, our results indicated that different PTMs in the spike protein RBD may result in distinctive binding amino acid sites when interacting with host factors such as ACE2, KREMEN1, and ASGR1. In other words, PTM might lead to profound structural changes in the interaction sites, which may further impact the virus’ virulence. These results may shed light on the design of effective therapeutic interventions based on high-resolution PTM-impacted protein structures and may aid drug discovery, antibody design, and the effective treatment of SARS-CoV-2 infection.

## Discussion

Overall, we characterized a total of 87 PTM sites on 5 major modification types in the SARS-CoV-2 spike protein; many of the PTMs are novel. Subsequently, we proposed and validated a computational approach to predict the spike protein’s structures from widely studied PTMs using in silico mutagenesis and Alphafold2. Results showed substantial changes in the spike protein’s structures, especially in RBD, as a result of amino acid modifications. For example, by replacing the phosphorylated amino acids with acidic amino acid, we observed tremendous changes in the RBD of the protein structure in comparison to the predicted structure from primary amino acid sequences and the experimental structure from cryo-EM experiments. Our study suggests that virulence is partially explained by the PTMs. For instance, different PTM types have varying impacts on the change in protein structure, with methylation having the largest effect and ubiquitination having the smallest effect. Finally, based on the post-translationally modified spike proteins, we further analyzed the binding affinity and intermolecular interactions between the SARS-CoV-2 spike protein and its host factor, including ACE2, ASGR1, and KREMEN1, to demonstrate that the PTMs can significantly affect the interaction between the spike protein and host factors. In summary, in the absence of costly and laborious methods for characterizing protein structural changes due to PTMs, we believe that our proposed innovative computational algorithm can pinpoint an effective approach for protein structural characterization and functional studies. Nevertheless, these results require further validation via experimental x-ray crystallography and/or cryo-EM.

## Materials and Methods

LC-MS/MS proteomics analyses by Proteome Discoverer (version 2.4) were employed to characterize the PTM landscape from raw spectrum data of the SARS-CoV-2 spike protein, followed by an in silico site-directed mutagenesis approach: amino acid substitution to replace post-translationally modified sites. Afterwards, the deep learning tool Alphafold2 was used to predict protein structure from the spike protein with replaced amino acids. To evaluate the impact of various PTMs on the spike protein, a head-to-tail comparison between the original unmodified sequence and PTM-modified sequences was conducted by Alphafold2. Finally, we examined the PTM’s role in the RBD region of the spike protein to reveal its interaction with host factors such as ACE2. The workflow in Fig. 1 described the detailed procedure in this study.

### Data access

We retrieved the primary amino acid sequence of the SARS-CoV-2 alpha strain spike protein from UniProt (accession no. P0DTC2), which is the first accurate sequence with no mutation and complete structural domain [38]. The protein’s 3D structure from the cryo-EM was obtained from Research Collaboratory for Structural Bioinformatics - Protein Data Bank (RCSB PDB) (ID: 6VSB and 6VXX) [24,25]. We also selected PDB ID: 6LZG as the structure of the spike protein RBD for the host factor ACE2 receptor [18,39]. Apart from that, 6 LC-MS/MS raw files (PXD023346 in the MassIVE proteomics database) were downloaded for downstream LC-MS/MS quantitative PTM identification.

### Construction of the high-resolution quantitative map of spike protein PTMs

To generate the PTM landscape and elucidate the role of PTM stoichiometry in the SARS-CoV-2 spike protein’s 3D structure, we analyzed a set of LC-MS/MS raw files by the Proteome Discoverer 2.4 software using the SARS-CoV-2 spike protein sequence from UniProt (accession no. P0DTC2), as shown in Fig. 1. Chymotrypsin, α-lytic endopeptidase, and LysC-trypsin were set as cleavage reagents based on the corresponding experiments. Up to 2 missed cleavages were allowed. A mass tolerance of 10 parts per million and 0.02 Da were set for the precursor and fragment ion, respectively. Cysteine carbamidomethylation was set as a fixed modification, while methionine oxidation and protein N-terminus acetylation were set as variable modifications. For comprehensive PTM analysis, phosphorylation (S/T/Y), acetylation (K), methylation (K/R/E), and ubiquitination (K) were individually investigated in parallel database searches for respective variable modification. A peptide-level false discovery rate of 1% was set to filter the result. The site probability threshold was set as 75. Finally, to further filter the modification sites, manual validation was performed. Apart from that, we also referred to the glycosylation PTMs of the SARS-CoV-2 spike protein in a previously published literature [1].

### In silico site-directed mutagenesis to derive substituted amino acid sequences on PTM sites

Mutagenesis is a popular strategy to study protein modifications by replacing an amino acid with another amino acid to understand how the consequence of a PTM in terms of residue change impacts the protein structure and function [32,33,37,40]. In experimental chemistry, scientists introduced specific nonsynonymous DNA sequence mutations that resulted in change in selected amino acids. In this study, we perform computational, in silico mutagenesis by directly replacing/substituting an amino acid with a different amino acid to mimic the experimental mutagenesis. As an example, the amino acids of the SARS-CoV-2 spike protein primary sequence (alpha) with clearly defined PTMs will be substituted through the site-directed mutagenesis approach that resulted in primary amino acid sequence changes. Importantly, these substitutions are strictly based on physicochemical property changes and have been extensively validated experimentally [1,8]. For instance, we substitute serine or threonine to aspartic acid, the negatively charged amino acid, to mimic phosphorylation [8,33]. Glycosylated modification with the pattern asparagine-X-serine/threonine (N-X-S/T, X is any amino acid except proline) can be mimicked by glutamic acid and alanine [40–43]. In addition, ubiquitination often occurs at the lysine site and will be substituted with arginine based on mutagenesis [44]. Methylation typically occurs in lysine or arginine, and lysine will be “mutated” (in silico substituted) to arginine, while arginine will be “mutated” to lysine [37,45]. For acetylation, the lysine residue will be mutated to arginine [32].

### Computational prediction of protein structure from amino acid sequences using Alphafold2

The modified amino acid sequences of the spike protein by the in silico mutagenesis substitution rule were submitted to AlphaFold2 v2.0 to analyze the impacts of PTMs on the spike protein structure [9,23]. In particular, we performed both monomer and multimer spike protein structure prediction. On the one hand, we wanted to focus on the individual spike protein sequence and describe how the PTM affects both the modified amino acids’ surrounding region and the overall protein structure based on the monomer prediction approach. On the other hand, we would like to evaluate how the spike protein binds to host factors such as ACE2, KREMEN1, and ASGR1 receptor [12,19,20]. All computations were conducted using a high-performance platform, including 4 Tesla A100 GPUs with 80 GB of memory, an Intel (R) Xeon(R) Gold 6348 CPU, and a 512-GB RAM. After completion, the output .pdb files were further analyzed.

### Evaluation of the impact of PTMs by aligning predicted spike protein structures influenced by different PTMs

The identification of significantly altered regions of the spike protein structure is critical to the understanding of virus evolution and transmission [46]. To this end, we comprehensively evaluated the impacts of PTMs on spike protein structure and demonstrated the capability of this method to review key structural differences. On the one hand, we used pLDDT, a per-residue measure of local confidence, on a scale from 0 to 100 by Alphafold2 to identify surrounding regions to reveal the degree of consistency within the spike protein structures that are heavily post-translationally modified and to determine whether the amino acid is accessible for binding [23,47]. Basically, regions with pLDDT above 70 are expected to be in good condition and those with pLDDT below 50 are considered as low confident. On the other hand, we also aligned the computational predicted structures to cryo-EM experiments (PDB ID: 6VSB and 6VXX) using PyMOL and the US-align method. Specifically, we performed the RMSD assessment of atomic positions. RMSD is one of the most widely used methods to evaluate the pairwise similarity between 2 protein structures, by calculating the average distance among atoms of superimposed proteins, expressed in ångströms (Å) (10^−10^m). Furthermore, template modeling-score (TM-score) was also introduced for multiple structure alignments, which takes values in the range of (0,1], where 1 indicates an identical structure match and a value greater than 0.45 means the structures share the same global topology for proteins [36].

### Computational prediction of the interaction affinity between the spike protein RBD and host factor receptor

We selected the RBD region (amino acid positions: 333 to 531) of the predicted spike protein structure and aligned this region to the cryo-EM experiment structure (PDB ID: 6LZG), a novel coronavirus spike RBD complex with its receptor ACE2 [18], and we generated protein–protein interaction complexes using PyMOL. Afterwards, we introduced the PRODIGY web server to predict the binding affinity in virus–host factor interaction complexes and identified key amino acids involved in the protein–protein interactions for each generated 3D structure [48,49]. Furthermore, we also calculated the number of ICs at the interacting complex interface with a thresholding distance of 5.5 Å. Importantly, the binding affinity (kcal mol^−1^) was implemented based on a linear regression using ICs and noninteracting surfaces of protein–protein complexes. Basically, the lower values (greater absolute values) indicate stronger binding affinity of protein–protein complexes [48]. In summary, this study allows us to find the crucial binding amino acids based on post-translationally modified structures to show that the PTM strongly affects the function of the spike protein, especially its binding to the ACE2 protein.

## Data Availability

All data supporting the findings of this study are available within the paper and its supplementary information.

## Supplementary Materials

Supplementary Materials Table S1. The distribution of 87 PTM sites from major modifications on the SARS-CoV-2 spike protein. Table S2. The binding affinity between the spike protein receptor-binding domain and host factor receptor.

## References

[1] Tian W, Li D, Zhang N, Bai G, Yuan K, Xiao H, Gao F, Chen Y, Wong CCL, Gao GF. O-glycosylation pattern of the SARS-CoV-2 spike protein reveals an “O-follow-N” rule. Cell Res. 2021;31(10):1123–1125. 34341488 10.1038/s41422-021-00545-2 PMC8326647

[2] Stukalov A, Girault V, Grass V, Karayel O, Bergant V, Urban C, Haas DA, Huang Y, Oubraham L, Wang A, et al. Multilevel proteomics reveals host perturbations by SARS-CoV-2 and SARS-CoV. Nature. 2021;594(7862):246–252. 33845483 10.1038/s41586-021-03493-4

[3] Yang Q, Syed AAS, Fahira A, Shi Y. Structural analysis of the SARS-CoV-2 omicron variant proteins. Research. 2021;2021:Article 9769586. 35088054 10.34133/2021/9769586 PMC8765807

[4] Plante JA, Liu Y, Liu J, Xia H, Johnson BA, Lokugamage KG, Zhang X, Muruato AE, Zou J, Fontes-Garfias CR, et al. Spike mutation D614G alters SARS-CoV-2 fitness. Nature. 2021;592:116–121. 33106671 10.1038/s41586-020-2895-3 PMC8158177

[5] Wu L, Peng C, Yang Y, Shi Y, Zhou L, Xu Z, Zhu W. Exploring the immune evasion of SARS-CoV-2 variant harboring E484K by molecular dynamics simulations. Brief Bioinform. 2022;23(1):Article bbab383. 34553217 10.1093/bib/bbab383 PMC8500006

[6] Sun C, Xie C, Bu G-L, Zhong L-Y, Zeng M-S. Molecular characteristics, immune evasion, and impact of SARS-CoV-2 variants. Signal Transduct Target Ther. 2022;7:Article 202. 35764603 10.1038/s41392-022-01039-2 PMC9240077

[7] Yang Q, Jian X, Syed AAS, Fahira A, Zheng C, Zhu Z, Wang K, Zhang J, Wen Y, Li Z, et al. Structural comparison and drug screening of spike proteins of ten SARS-CoV-2 variants. Research. 2022;2022:Article 9781758. 35198984 10.34133/2022/9781758 PMC8829538

[8] Barber KW, Rinehart J. The ABCs of PTMs. Nat Chem Biol. 2018;14:188–192. 29443972 10.1038/nchembio.2572 PMC5979263

[9] Bagdonas H, Fogarty CA, Fadda E, Agirre J. The case for post-predictional modifications in the AlphaFold protein structure database. Nat Struct Mol Biol. 2021;28:869–870. 34716446 10.1038/s41594-021-00680-9

[10] Bludau I, Willems S, Zeng WF, Strauss MT, Hansen FM, Tanzer MC, Karayel O, Schulman BA, Mann M. The structural context of posttranslational modifications at a proteome-wide scale. PLoS Biol. 2022;20(5):Article e3001636. 35576205 10.1371/journal.pbio.3001636 PMC9135334

[11] Fung TS, Liu DX. Post-translational modifications of coronavirus proteins: Roles and function. Future Virol. 2018;13(6):405–430. 32201497 10.2217/fvl-2018-0008 PMC7080180

[12] Kapoor K, Chen T, Tajkhorshid E. Posttranslational modifications optimize the ability of SARS-CoV-2 spike for effective interaction with host cell receptors. Proc Natl Acad Sci USA. 2022;119(28):Article e2119761119. 35737823 10.1073/pnas.2119761119 PMC9282386

[13] Adams C, Boonen K, Laukens K, Bittremieux W. Open modification searching of SARS-CoV-2–human protein interaction data reveals novel viral modification sites. Mol Cell Proteomics. 2022;100425(12):Article 100425. 10.1016/j.mcpro.2022.100425 PMC9554009 36241021

[14] Supekar NT, Shajahan A, Gleinich AS, Rouhani DS, Heiss C, Chapla DG, Moremen KW, Azadi P. Variable posttranslational modifications of severe acute respiratory syndrome coronavirus 2 nucleocapsid protein. Glycobiology. 2021;31(9):1080–1092. 33997890 10.1093/glycob/cwab044 PMC8241430

[15] Lan J, Ge J, Yu J, Shan S, Zhou H, Fan S, Zhang Q, Shi X, Wang Q, Zhang L, et al. Structure of the SARS-CoV-2 spike receptor-binding domain bound to the ACE2 receptor. Nature. 2020;581:215–220. 32225176 10.1038/s41586-020-2180-5

[16] Yang J, Petitjean SJL, Koehler M, Zhang Q, Dumitru AC, Chen W, Derclaye S, Vincent SP, Soumillion P, Alsteens D. Molecular interaction and inhibition of SARS-CoV-2 binding to the ACE2 receptor. Nat Commun. 2020;11:Article 4541. 32917884 10.1038/s41467-020-18319-6 PMC7486399

[17] Hoffmann M, Kleine-Weber H, Schroeder S, Krüger N, Herrler T, Erichsen S, Schiergens TS, Herrler G, Wu NH, Nitsche A, et al. SARS-CoV-2 cell entry depends on ACE2 and TMPRSS2 and is blocked by a clinically proven protease inhibitor. Cell. 2020;181(2):271–280.e8. 32142651 10.1016/j.cell.2020.02.052 PMC7102627

[18] Wang Q, Zhang Y, Wu L, Niu S, Song C, Zhang Z, Lu G, Qiao C, Hu Y, Yuen KY, et al. Structural and functional basis of SARS-CoV-2 entry by using human ACE2. Cell. 2020;181(4):894–904.e9. 32275855 10.1016/j.cell.2020.03.045 PMC7144619

[19] Gu Y, Cao J, Zhang X, Gao H, Wang Y, Wang J, He J, Jiang X, Zhang J, Shen G, et al. Receptome profiling identifies KREMEN1 and ASGR1 as alternative functional receptors of SARS-CoV-2. Cell Res. 2022;32(1):24–37. 34837059 10.1038/s41422-021-00595-6 PMC8617373

[20] Hoffmann M, Pöhlmann S. Novel SARS-CoV-2 receptors: ASGR1 and KREMEN1. Cell Res. 2022;32:1–2. 34903854 10.1038/s41422-021-00603-9 PMC8666617

[21] Olzscha H. Posttranslational modifications and proteinopathies: How guardians of the proteome are defeated. Biol Chem. 2019;400(7):895–915. 30998500 10.1515/hsz-2018-0458

[22] Zeng J, Jiang F, Wu YD. Mechanism of phosphorylation-induced folding of 4E-BP2 revealed by molecular dynamics simulations. J Chem Theory Comput. 2017;13(1):320–328. 28068774 10.1021/acs.jctc.6b00848

[23] Jumper J, Evans R, Pritzel A, Green T, Figurnov M, Ronneberger O, Tunyasuvunakool K, Bates R, Žídek A, Potapenko A, et al. Highly accurate protein structure prediction with AlphaFold. Nature. 2021;596:583–589. 34265844 10.1038/s41586-021-03819-2 PMC8371605

[24] Wrapp D, Wang N, Corbett KS, Goldsmith JA, Hsieh CL, Abiona O, Graham BS, McLellan JS. Cryo-EM structure of the 2019-nCoV spike in the prefusion conformation. Science. 2020;367(6483):1260–1263. 32075877 10.1126/science.abb2507 PMC7164637

[25] Walls AC, Park YJ, Tortorici MA, Wall A, McGuire AT, Veesler D. Structure, function, and antigenicity of the SARS-CoV-2 spike glycoprotein. Cell. 2020;181(2):281–292.e6. 32155444 10.1016/j.cell.2020.02.058 PMC7102599

[26] Wang D, Liu D, Yuchi J, He F, Jiang Y, Cai S, Li J, Xu D. MusiteDeep: A deep-learning based webserver for protein post-translational modification site prediction and visualization. Nucleic Acids Res. 2021;48(W1):W140–W146. 10.1093/nar/gkaa275 PMC7319475 32324217

[27] Liu Y, Wang M, Xi J, Luo F, Li A. PTM-ssMP: A web server for predicting different types of post-translational modification sites using novel site-specific modification profile. Int J Biol Sci. 2018;14(8):946–956. 29989096 10.7150/ijbs.24121 PMC6036757

[28] Ford CT, Jacob Machado D, Janies DA. Predictions of the SARS-CoV-2 omicron variant (B.1.1.529) spike protein receptor-binding domain structure and neutralizing antibody interactions. Front Virol. 2022;2:Article 830202.

[29] Qiu Y, Zhao Y-B, Wang Q, Li J-Y, Zhou Z-J, Liao C-H, Ge X-Y. Predicting the angiotensin converting enzyme 2 (ACE2) utilizing capability as the receptor of SARS-CoV-2. Microbes Infect. 2020;22(4–5):221–225. 32199943 10.1016/j.micinf.2020.03.003 PMC7156207

[30] Chen C, Boorla VS, Banerjee D, Chowdhury R, Cavener VS, Nissly RH, Gontu A, Boyle NR, Vandegrift K, Nair MS, et al. Computational prediction of the effect of amino acid changes on the binding affinity between SARS-CoV-2 spike RBD and human ACE2. Proc Natl Acad Sci USA. 2021;118(42):Article e2106480118. 34588290 10.1073/pnas.2106480118 PMC8594574

[31] Yan F-F, Gao F. Comparison of the binding characteristics of SARS-CoV and SARS-CoV-2 RBDs to ACE2 at different temperatures by MD simulations. Brief Bioinform. 2021;22(2):1122–1136. 33611368 10.1093/bib/bbab044 PMC7929385

[32] Yang A, Cho K, Park HS. Chemical biology approaches for studying posttranslational modifications. RNA Biol. 2018;15(4–5):427–440. 28901832 10.1080/15476286.2017.1360468 PMC6103722

[33] Pearlman SM, Serber Z, Ferrell JE Jr. A mechanism for the evolution of phosphorylation sites. Cell. 2011;147(4):934–946. 22078888 10.1016/j.cell.2011.08.052 PMC3220604

[34] Ali I, Conrad RJ, Verdin E, Ott M. Lysine acetylation goes global: From epigenetics to metabolism and therapeutics. Chem Rev. 2018;118(3):1216–1252. 29405707 10.1021/acs.chemrev.7b00181 PMC6609103

[35] McCallum M, De Marco A, Lempp FA, Tortorici MA, Pinto D, Walls AC, Beltramello M, Chen A, Liu Z, Zatta F, et al. N-terminal domain antigenic mapping reveals a site of vulnerability for SARS-CoV-2. Cell. 2021;184(9):2332–2347.e16. 33761326 10.1016/j.cell.2021.03.028 PMC7962585

[36] Zhang C, Shine M, Pyle AM, Zhang Y. US-align: Universal structure alignments of proteins, nucleic acids, and macromolecular complexes. Nat Methods. 2022;19:1109–1115. 36038728 10.1038/s41592-022-01585-1

[37] Guccione E, Richard S. The regulation, functions and clinical relevance of arginine methylation. Nat Rev Mol Cell Biol. 2019;20:642–657. 31350521 10.1038/s41580-019-0155-x

[38] Wu F, Zhao S, Yu B, Chen Y-M, Wang W, Song Z-G, Hu Y, Tao Z-W, Tian J-H, Pei Y-Y, et al. A new coronavirus associated with human respiratory disease in China. Nature. 2020;579:265–269. 32015508 10.1038/s41586-020-2008-3 PMC7094943

[39] Hussain M, Jabeen N, Raza F, Shabbir S, Baig AA, Amanullah A, Aziz B. Structural variations in human ACE2 may influence its binding with SARS-CoV-2 spike protein. J Med Virol. 2020;92(9):1580–1586. 32249956 10.1002/jmv.25832 PMC7228372

[40] Schwarz F, Aebi M. Mechanisms and principles of N-linked protein glycosylation. Curr Opin Struct Biol. 2011;21(5):576–582. 21978957 10.1016/j.sbi.2011.08.005

[41] Davis BG. Sugars and proteins: New strategies in synthetic biology. Pure Appl Chem. 2009;81(2):285–298.

[42] Varki A, Cummings RD, Esko JD, Stanley P, Hart GW, Aebi M, Darvill AG, Kinoshita T, Packer NH, Prestegard JH, et al., editors. *Essentials of glycobiology [internet]*. Cold Spring Harbor (NY): Cold Spring Harbor Laboratory Press; 2015. 27010055

[43] Ressler VT, Raines RT. Consequences of the endogenous *N*-glycosylation of human ribonuclease 1. Biochemistry. 2019;58(7):987–996. 30633504 10.1021/acs.biochem.8b01246 PMC6380942

[44] Petroski MD, Deshaies RJ. Mechanism of lysine 48-linked ubiquitin-chain synthesis by the cullin-RING ubiquitin-ligase complex SCF-Cdc34. Cell. 2005;123(6):1107–1120. 16360039 10.1016/j.cell.2005.09.033

[45] Blanc RS, Richard S. Arginine methylation: The coming of age. Mol Cell. 2017;65(1):8–24. 28061334 10.1016/j.molcel.2016.11.003

[46] Waman VP, Sen N, Varadi M, Daina A, Wodak SJ, Zoete V, Velankar S, Orengo C. The impact of structural bioinformatics tools and resources on SARS-CoV-2 research and therapeutic strategies. Brief Bioinform. 2021;22(2):742–768. 33348379 10.1093/bib/bbaa362 PMC7799268

[47] Mariani V, Biasini M, Barbato A, Schwede T. IDDT: A local superposition-free score for comparing protein structures and models using distance difference tests. Bioinformatics. 2013;29(21):2722–2728. 23986568 10.1093/bioinformatics/btt473 PMC3799472

[48] Xue LC, Rodrigues JP, Kastritis PL, Bonvin AM, Vangone A. PRODIGY: A web server for predicting the binding affinity of protein–protein complexes. Bioinformatics. 2016;32(23):3676–3678. 27503228 10.1093/bioinformatics/btw514

[49] Vangone A, Bonvin AMJJ. Contacts-based prediction of binding affinity in protein–protein complexes. eLife. 2015;4:Article e07454. 26193119 10.7554/eLife.07454 PMC4523921

